# A Rare Case of Allantoic Cyst with Patent Urachus in Fetus with a Microdeletion in 1q21.1q21.2 Region

**DOI:** 10.3390/diagnostics11122332

**Published:** 2021-12-11

**Authors:** Alexandra Bouariu, Ana Maria Scutelnicu, Anca Marina Ciobanu, Brîndușa Ana Cimpoca Raptis, Andreea Elena Dumitru, Florina Nedelea, Nicolae Gică, Anca Maria Panaitescu

**Affiliations:** 1 Filantropia Clinical Hospital Bucharest, 011117 Bucharest, Romania; alexandra.bouariu@yahoo.com (A.B.); ana.scutelnicu@yahoo.com (A.M.S.); ciobanu.ancamarina@gmail.com (A.M.C.); brindusa.cimpoca@gmail.com (B.A.C.R.); helena_dumitru@yahoo.com (A.E.D.); ina.nedelea@gmail.com (F.N.); gica.nicolae@umfcd.ro (N.G.); 2 Department of Obstetrics and Gynecology, “Carol Davila” University of Medicine and Pharmacy, 020021 Bucharest, Romania; 3 Department of Human Genetics, Clinical Hospital Filantropia Bucharest, 011117 Bucharest, Romania

**Keywords:** allantoic cyst, umbilical cyst, fetal bladder, fetal ultrasound

## Abstract

An allantoic cyst is a rare malformation with a frequency of 3 in 1,000,000 that may be seen antenatally by ultrasound assessment when the connection between the cloaca (future bladder) and the allantois fails to regress. A patent urachus that presents as a cyst (allantoic) is usually considered not to be associated with chromosomal abnormalities, but if it is not repaired after birth this leads to complications such as urinary tract infections and stone formation. We present a case of a fetus diagnosed with allantoic cyst at the first trimester ultrasound assessment at 12 weeks gestation. The follow up scans showed a decrease in size of the allantoic cyst with no other obvious major defects and, when invasive testing (amniocentesis with microarray analysis) was performed, a rare microdeletion, 1q21.1q21.2 was identified (1.82 Mb deletion).

**Figure 1:**
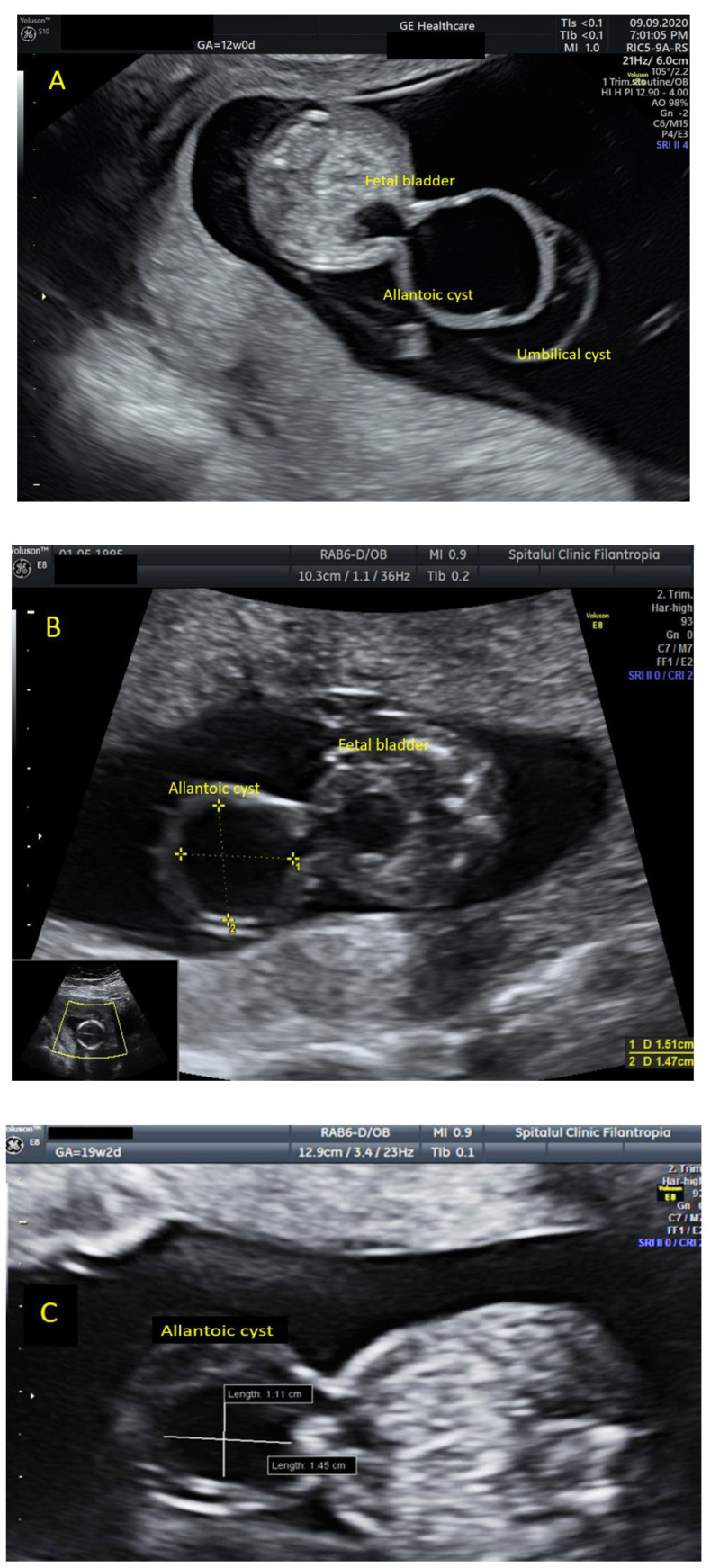

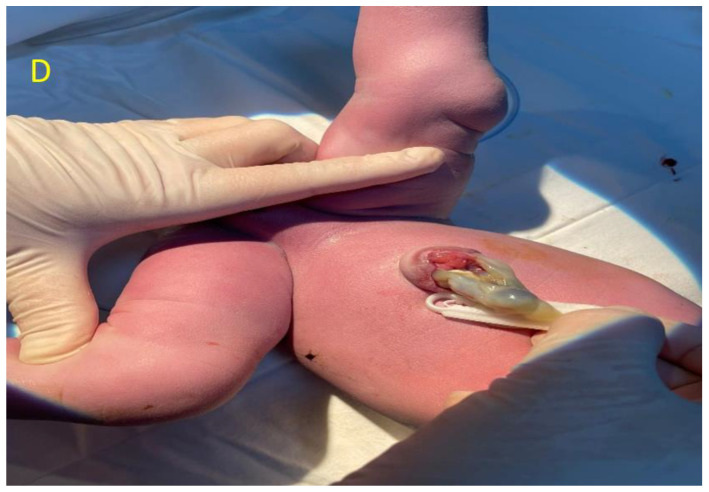
We present a case of a 25-year-old G3P1, fit and healthy, who presented to our unit in her first trimester for the combined screening test assessment [1,2]. The early ultrasound assessment of the fetal anatomy demonstrated a cystic structure at the level of fetal cord insertion that was connected to fetal bladder, measuring 21.1 × 23.1 mm (**A**). The suspicion of allantoic cyst [3] was raised due to the patent urachus demonstrated on the ultrasound assessment [4]. An additional umbilical cyst was seen next to the allantoic cyst. Differential diagnosis with umbilical cord cyst, omphalocele and bladder exstrophy has been considered [5]. No other obvious major abnormalities have been seen and the nuchal translucency was within normal limits with a low chance for common trisomies (T21/T13/T18) from the combined screening test. A follow up scan at 16 weeks showed similar findings with the previous scan, the cystic structure keeping smaller measurements (14.7 × 15.1 mm) (**B**). The anomaly scan at 19 weeks demonstrated no other structural anomalies beside the cystic structure that was measuring smaller than before (11.1 × 14.5 mm) (**C**). In view of this findings, the mother chose to have invasive testing (amniocentesis), and the result of microarray showed 1.82 Mb microdeletion in the 1q21.1q21.2 region. The consequences of 1q21.1 microdeletion may include mild developmental delay, dysmorphic craniofacial features, microcephaly, eye abnormalities, cardiac defects, brain malformations as hydrocephalus and agenesis of the corpus callosum [6,7]. Genitourinary anomalies as vesicoureteral reflux, hydronephrosis, inguinal hernia, and cryptorchidism can be present [8]. Because the clinical spectrum is broad and persons with deletion do not have obvious clinical findings, the family decided to continue the pregnancy [9]. In order to establish the familial or “de novo” origin of this microdeletion, parental testing (microarray/qPCR) is advised [10]. The pregnancy evolved without complications and the third trimester scan showed small measurements of the cystic structure. A live neonate was delivered at 39 weeks by elective caesarean, weighing 3000 g (**D**). The neonate underwent surgical repair of the abdominal wall in the first weeks of life and no other complications have been reported during postnatal care. We have followed up the child within the first year of life and no dysmorphic craniofacial features, microcephaly, or renal disorders have been reported. In terms of brain development, the baby achieved the expected milestones for the first year of life including rolling over, sitting up, standing, and initially walking.
